# Genomic Comparison of Eight Closed Genomes of Multidrug-Resistant *Salmonella enterica* Strains Isolated From Broiler Farms and Processing Plants in Trinidad and Tobago

**DOI:** 10.3389/fmicb.2022.863104

**Published:** 2022-05-10

**Authors:** Meghan Maguire, Anisa S. Khan, Abiodun A. Adesiyun, Karla Georges, Narjol Gonzalez-Escalona

**Affiliations:** ^1^Division of Microbiology, Office of Regulatory Science, Center for Food Safety and Applied Nutrition, Food and Drug Administration, College Park, MD, United States; ^2^School of Veterinary Medicine, Faculty of Medical Sciences, University of the West Indies, St. Augustine, Trinidad and Tobago

**Keywords:** *Salmonella*, multi-drug resistance, complete genome, nanopore sequencing, poultry, Trinidad and Tobago

## Abstract

*Salmonella enterica* is an important foodborne pathogen worldwide. We used long and short-read sequencing to close genomes of eight multidrug-resistant (MDR) *S. enterica* strains, belonging to serovars Infantis (2), Albany, Oranienburg, I 4,[5],12:i:-, Javiana, Schwarzengrund, and Kentucky from broiler chicken farms and processing plants in Trinidad and Tobago. They also belonged to seven different sequence types (STs- 32, 292, 1510, 19, 24, 152, and 96). Among the strains, seven had demonstrated multi-drug resistance with the presence of at least three AMR genes, whereas three isolates contained the quinolone resistance gene *qnr*_B19_ in plasmids (CFSAN103840, CFSAN103854, and CFSAN103872). The extended-spectrum β-lactamase genes *bla*_CTX−M−65_ (CFSAN103796) and *bla*_TEM−1_ (CFSAN103852) were detected in this study. The genomes closed in this study will be useful for future source tracking and outbreak investigations in Trinidad and Tobago and worldwide.

## Significance

*Salmonella enterica* contamination of poultry is a major route of transmission to humans. We have closed genomes of multiple *Salmonella* serovars and report multiple antimicrobial resistance (AMR) genes from isolates collected in processing plants and retail markets in Trinidad and Tobago. These closed genomes will aid in monitoring AMR transmission and future outbreak investigations.

## Introduction

*Salmonella enterica* is one of the most important foodborne pathogens in the world (Gonzalez-Escalona et al., 2009). There are more than 325,000 *Salmonella* genomes in NCBI encompassing isolates from many countries (https://www.ncbi.nlm.nih.gov/pathogens/). Non-typhoidal *Salmonella* (NTS) is responsible for ~93,757,000 cases of gastroenteritis and 155,000 deaths worldwide each year. Nearly 86% are linked to foodborne transmission (Majowicz et al., 2010). Poultry, including eggs and egg products, is one of the main reservoirs of *Salmonella* and has often been implicated as the source of many outbreaks worldwide (Schoeni et al., 1995; Kuehn, 2010; Jackson et al., 2013; Deng et al., 2014; Antunes et al., 2016).

*Salmonella* serovars Enteritidis, Newport, Typhimurium, Javiana, I 4,[5],12:i:-, and Heidelberg have been among the top five serovars associated with human illness over the last 15 years (2006–2016) in the United States (https://www.cdc.gov/nationalsurveillance/pdfs/2016-Salmonella-report-508.pdf). *S*. serovar Infantis has shown the highest percent increase in reported cases over the same time frame (+168.5%). While, *S*. serovar Kentucky has become the most commonly detected serovar in chicken (Foley et al., 2011), but is not among the top 20 serovars causing human illness.

While most *Salmonella* infections result in gastroenteritis without the need for additional medical intervention, severe, invasive infections can require antimicrobial treatment. Multidrug resistance (MDR) in *Salmonella* is closely monitored in clinical and environmental isolates around the world [National Antimicrobial Resistance Monitoring System (NARMS), European Center for Disease Control and Prevention (ECDC), and European Food Safety Authority (EFSA)]. In the United States, the FDA has issued guidance on the judicious use of antimicrobial agents in food-producing animals (https://www.fda.gov/regulatory-information/search-fda-guidance-documents/cvm-gfi-209-judicious-use-medically-important-antimicrobial-drugs-food-producing-animals) and individual antimicrobial agent restrictions have resulted in the decreased prevalence of resistance-conferring genes (https://www.fda.gov/media/108304/download).

Several antimicrobial-resistant (AMR) *Salmonella* strains were isolated during a *Salmonella* and AMR surveillance program of poultry production facilities in Trinidad and Tobago from 2016 to 2019 (Khan et al., 2022b). Their genomes were sequenced by short-read sequencing, and we selected a group of eight strains belonging to multiple serovars and showing a diverse range of MDR, to completely close their genomes using a combination of long-read and short-read sequencing technologies. The availability of these closed genomes will be useful for future outbreak investigations and for understanding AMR acquisition and transmission in *Salmonella* within the local Trinidad and Tobago region and worldwide.

## Materials and Methods

### Bacterial Strains and Media

Cloacal swabs, drag swabs, whole chicken carcasses, and neck skin samples were collected from retail outlets, processing plants, and farms in Trinidad and Tobago during an AMR surveillance program from 2016 to 2019. The sample was pre-enriched in buffered peptone water (Oxoid, Ltd., Hampshire, UK) for 18–24 h at 37°C, then selectively enriched in tetrathionate broth (Oxoid), and thereafter incubated for 18–24 h at 37°C. The sample enriched in selective broth was then sub-cultured onto xylose lysine Tergitol 4 (Oxoid) and incubated aerobically at 37°C for 18–24 h. Suspected *Salmonella* colonies that displayed characteristic colonies on the selective agar plate were then purified on blood agar plates (Oxoid) and incubated at 37°C for 18–24 h. Pure cultures were subjected to a panel of biochemical tests that included triple sugar iron agar, lysine iron agar, urea, citrate, methyl red, sulfide-indole-motility medium, and o-nitrophenyl-b-D-galactopyranoside (Oxoid) (Andrews, 1992). Biochemically confirmed isolates were then subjected to serological typing by using *Salmonella* polyvalent antiserum (A-I and Vi, Difco, Detroit, MI). Complete confirmation and serotyping of *Salmonella* isolates were performed by using the phase reversal technique, and the results were interpreted according to the Kauffman-White scheme (https://www.pasteur.fr/sites/default/files/veng_0.pdf) at the Public Health Laboratory, Ministry of Health, St. Michael, Barbados. The single isolate strains were grown overnight in tryptic soy broth (TSB) medium at 35°C. The strains to have their genomes entirely closed were selected among 146 strains isolated by Khan et al. (2022b). The criteria employed for their selection was: (1) a representative of each predominant serovar from that study [Kentucky 20.5% (30/146), Javiana 19.2% (28/146), Infantis 13.7% (20/146), and Albany 8.9% (13/146)], (2) Infantis with a different AMR profile, and 3) uncommon serotypes isolated from poultry [Oranienburg, I 4,[5],12:i:- (a monophasic variant of *Salmonella* Typhimurium), and Schwarzengrund].

### Serotyping and Antimicrobial Resistance Determination

Conventional serotyping methods using the phase reversal technique described previously (Khan et al., 2021, 2022a) were performed at the Public Health Laboratory, Ministry of Health, St. Michael, Barbados. The antimicrobial resistance was determined by the disk diffusion method as described previously (Khan et al., 2021, 2022a). Data generated from these two methods were compared to the genomic data.

### Nucleic Acid Extraction

DNA was extracted using the Maxwell RSC cultured cells DNA kit with a Maxwell RSC instrument (Promega, Madison, WI, USA) following the manufacturer's protocols for Gram-negative bacteria with additional RNase treatment. DNA concentration was determined by the Qubit 4 Fluorometer (Invitrogen, Carlsbad, CA, USA) according to the manufacturer's instructions.

### Illumina MiSeq Short-Read Sequencing

The short reads whole-genome sequence for this strain was generated by Illumina MiSeq sequencing with the MiSeq V3 kit using 2 × 250 bp paired-end chemistry (Illumina, San Diego, CA, USA) according to the manufacturer's instructions. The libraries were constructed using 100 ng of genomic DNA using the Illumina® DNA Prep (M) Tagmentation (Illumina), according to the manufacturer's instructions. Reads were trimmed with Trimmomatic version.36 (Bolger et al., 2014).

### Oxford Nanopore Long-Read Sequencing

The long reads for each strain were generated through GridION sequencing (Nanopore, Oxford, UK). The sequencing library was prepared using the rapid barcoding sequencing kit (SQK-RBK004). Each library was run in an FLO-MIN106 (R9.4.1) flow cell, according to the manufacturer's instructions, for 48 h. Default parameters were used for all software unless otherwise specified. The run was base called live with default settings (MinKNOW Core version 3.6.5, Bream version 4.3.16, and Guppy version 3.2.10). Reads <4000 bp and quality scores of <7 were discarded for downstream analysis, for an estimated genome average coverage of 59 – 250X.

### Contig Assembly and Annotation

The final complete genome [chromosome and plasmid(s), when present] for each strain was obtained using a previously described pipeline (Gonzalez-Escalona et al., 2018) with modifications. The final genomes were achieved by a hybrid assembly generated using both nanopore and MiSeq data with Unicycler version 4.8 (Wick et al., 2017). A second *de novo* genome assembly was obtained with Flye version 2.8 (Kolmogorov et al., 2019) using nanopore data. Both assemblies (Flye and Unicycler) for each strain were aligned with Mauve version 2.4 (Darling et al., 2004). If the two aligned assemblies agreed in synteny and size, then the Unicycler hybrid assembly was used as the final assembly (i.e., complete genome). Unicycler assembled the chromosomes and plasmids as circular closed and oriented the chromosomes to start at the *dnaA* gene. The genomes were annotated using the NCBI Prokaryotic Genomes Automatic Annotation Pipeline (PGAAP, http://www.ncbi.nlm.nih.gov/genome/annotation_prok) (Tatusova et al., 2016). *In silico* analysis was used to determine the multi-locus sequence type (MLST) with the MLST website for *Salmonella* (http://enterobase.warwick.ac.uk/species/index/senterica), serotype using SeqSero (Zhang et al., 2015) (http://www.denglab.info/Seqero) which infers the serovar from antigenic structure, and antimicrobial genes were found using ResFinder-4.1 (https://cge.cbs.dtu.dk/services/ResFinder/) (Bortolaia et al., 2020). *In silico* virulence profile was determined using the software Ridom Seqsphere+ version 6.0.2 (Ridom GmbH, Germany) and their task tool that uses the virulence factor database (VFDB) as a reference (Liu et al., 2019) to assign virulence factors.

### Genomic Data Analysis

To analyze the relationships among the eight NTS closed genomes we performed a core genome MLST (cgMLST) using Ridom Seqsphere+ version 6.0.2 (Ridom) as previously described (Toro et al., 2016). The genome of *S*. Enteritidis P125109 (NC_011294) was used as a reference genome (4,160 genes) (Thomson et al., 2008). The *S*. Enteritidis genome from strain EC20121175 (CP007269.2) was used for comparison with the reference genome to establish a list of core and accessory genome genes. Genes that are repeated in more than one copy in any of the two genomes were removed from the analysis as failed genes. A task template was created that contains both core and accessory genes for this reference SE strain for any future testing. Each individual locus (core or accessory genes) from strain P125109 was assigned allele number 1. The assemblies for each individual NTS closed genome in this study were queried against the task template and if the locus was found and was different from the reference genome or any other queried genome already in the database, a new number was assigned to that locus, and so on. After eliminating any loci that were missing from the genome of any strain used in our analyses, we performed the cgMLST analysis. These remaining loci were considered the core genome shared by the analyzed strains. We used Nei's DNA distance method (Nei et al., 1983) for calculating the matrix of genetic distance, taking only the number of same/different alleles in the core genes into consideration. A neighbor-joining (NJ) tree using the appropriate genetic distances was built after the cgMLST analysis.

### Plasmid Comparison

A comparison of the CFSAN103796 *bla*_CTX−M−65_-positive plasmid with the ones reported in Tate et al. (2017) was performed and visualized with CGView (https://proksee.ca/) (Stothard and Wishart, 2005).

### Data Deposition

The complete genome sequences of the eight *S. enterica* strains have been deposited at GenBank under the accession listed in Table 1.

**Table 1:** Metadata for the eight *Salmonella enterica* strains isolated from chicken on poultry farms and processing plants were reported in this study.

**CFSAN No**.	**Accession No. chromosome and plasmid(s) (size bp)**	**GC content (%)**	**Hybrid assembly genome coverage (X)**	**Illumina SRAs**	**Nanopore SRAs**	**AMR genes^a^**	**ST^b^**	**Serotype^c^**
				**(Read no.)**	**(Read no.)**			
CFSAN103796	CP066335 (4,727,835)	52.3	95	SRR11660184 (2,096,092)	SRR13073499 (47,009)	*mdsA, mdsB, mdsC, aac(6')-Iaa*	32	Infantis
	CP066336 (312,952)	50.3	83			*tetA, aph(3”), sul1, aadA1, aac(3)-Iva, aph(4)-Ia, blaCTX-M-65, dfrA14, qacEdelta1*		
CFSAN103854	CP066333 (4,944,981)	52.2	133	SRR11660185 (2,482,592)	SRR13073498 (61,306)	*mdsA, mdsB, mdsC, aac(6')-Iaa*	292	Albany
	CP066334 (3,071)	48.2	33			*qnrB19*		
CFSAN103872	CP066260 (5,053,975)	52.2	80	SRR11660187 (1,533,662)	SRR13073497 (41,126)	*mdsA, mdsB, mdsC, aac(6')-Iaa*	1510	Oranienburg^d^
	CP066261 (49,044)	49.3	265			*qnrB19, aph(3”)-Ib, aph(6)-Id*		
	CP066262 (5,320)	52.4	400					
CFSAN103852	CP066328 (4,836,001)	52.2	129	SRR12995791 (2,606,746)	SRR13073496 (70,597)	*mdsA, mdsB, mdsC, aac(6')-Iaa*	19	I 4,[5],12:i:- (Typhimurium)
	CP066329 (110,489)	50.2	189			*aph(3”)-Ib, aph(6)-Id, blaTEM-1*		
	CP066330 (93,844)	53.1	228					
	CP066331 (33,659)	41.3	310					
	CP066332 (1,749)	57.5	1400					
CFSAN103840	CP066325 (4,554,367)	52.1	105	SRR12995802 (2,110,046)	SRR13073495 (63,239)	*mdsA, mdsB, mdsC, aac(6')-Iaa*	24	Javiana
	CP066326 (2,579)	50.5	1000			*qnrB19*		
	CP066327 (1,395)	55.2	3000					
CFSAN103816	CP066324 (4,703,182)	52.3	59	SRR12995817 (2,060,084)	SRR13073494 (53,365)	*mdsA, mdsB, mdsC, aac(6')-Iaa*	32	Infantis
CFSAN103862	CP066321 (4,847,947)	52.2	125	SRR12995807 (1,866,844)	SRR13073493 (106,256)	*mdsA, mdsB, mdsC, aac(6')-Iaa*	96	Schwarzengrund
	CP066322 (144,839)	49.0	198			*aph(3”)-Ib, aph(6)-Id, sitABCD*		
	CP066323 (4,050)	42.8	700					
CFSAN103828	CP066318 (4,767,999)	52.2	30	SRR12995770 (1,746,768)	SRR13073492 (30,574)	*mdsA, mdsB, mdsC, aac(6')-Iaa*	152	Kentucky
	CP066319 (146,829)	49.4	48			*sitABCD, tetB, aph(3”)-Ib, aph(6)-Id*		
	CP066320 (46,120)	42.8	150					

a *In silico antimicrobial genes were found using ResFinder-4.1 (https://cge.cbs.dtu.dk/services/ResFinder/).*

b *In silico MLST used the MLST website for Salmonella (http://enterobase.warwick.ac.uk/species/index/senterica).*

c *In silico serotyping using SeqSero (Zhang et al., 2015) (http://www.denglab.info/SeqSero), a tool to infer serovar from the genes that determine the antigenic structure.*

d *Othmarschen by phenotypic method*.

## Results and Discussion

An AMR surveillance program of poultry farms and retail outlets in Trinidad and Tobago from 2016 to 2019 produced several *Salmonella* isolates that were confirmed to bsse *Salmonella* by selective microbiological plating. Initial sequencing efforts identified the sequence type (ST) and serovar for each sample by *in silico* analysis. We selected eight samples from multiple serovars to completely close genomes using a hybrid assembly with Illumina MiSeq short-read and Oxford Nanopore long-read sequencing. The short-read sequencing for these eight samples was performed over three sequencing runs with an average output of 12.6 Gb and estimated average genome coverage of 50–150 ×. The multiplexed nanopore sequencing for eight samples was carried out over two sequencing runs. The output for run 1 was 7.69 Gb (2.33 million reads with a quality score of 9.48 and N50−11,835 bp) and for running 2 was 3.71 Gb (694 thousand reads with a quality score of 9.93 and N50−12,930 bp). The estimated average genome coverage was 59–250 × (Table 1).

**Figure 1:**
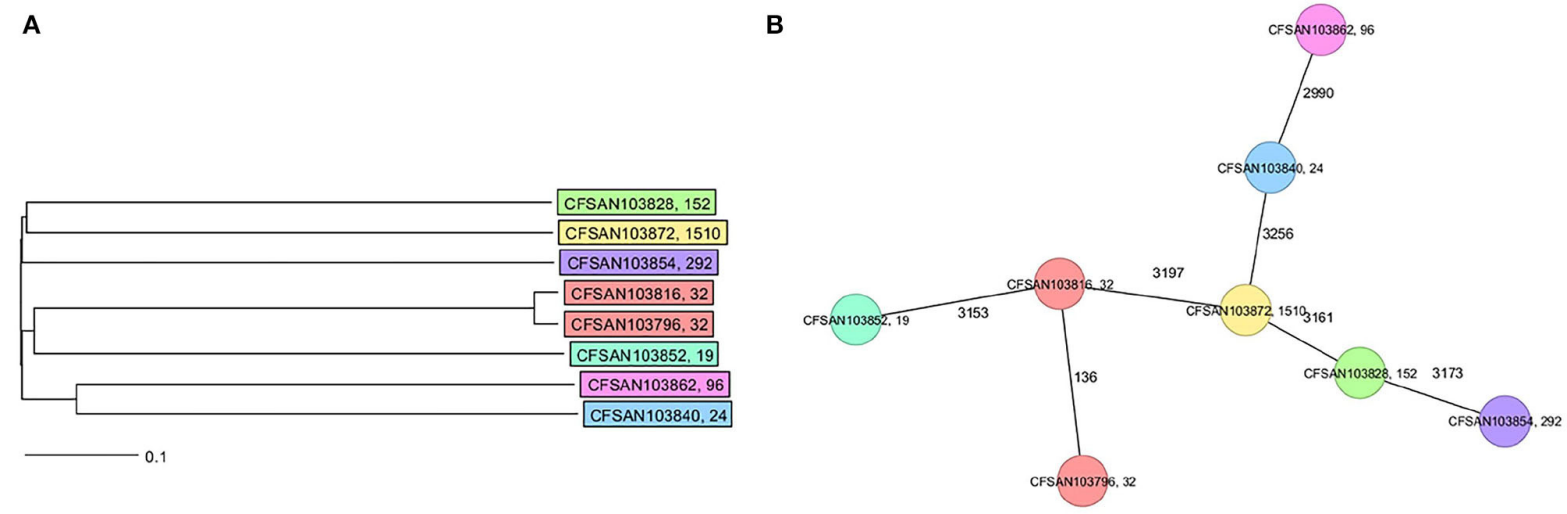
Core genome multi-locus sequence type (cgMLST) analysis of the eight *Salmonella* closed genomes based on 4,028 shared genes. **(A)** NJ tree showing the genetic differences among these genomes based on the shared loci. **(B)** Minimum spanning tree allowing to visualize the number of loci differences between these genomes. Strains are color-coded according to their serotype (ST). The ST is displayed after the name of the strain, separated by a comma. Numbers by lines represent the number of loci differing between strains. The lines are not drawn to scale.

The final closed genomes were generated by a hybrid assembly (Unicycler), which matched in synteny and size to a *de novo* assembly generated using only nanopore reads by the Flye assembler. *In silico* analysis confirmed the ST and serovar for each closed genome observed with the preliminary analysis run using the short-read sequencing (Khan et al., 2022b) (Table 1). The strains represented seven different STs and serotypes (32—Infantis, 292—Albany, 1,510—Oranienburg, 19−1,4,[5],12:i:- (Typhimurium), 24—Javiana, 96—Schwarzengrund and 152—Kentucky). There was an 88% agreement (7/8) in the serotyping results by phenotypic and *in silico* WGS (SeqSero) methods (Table 1). Strain CFSAN103872 was identified as Othmarschen by the phenotypic method but as Oranienburg by SeqSero. This discrepancy can be explained by the fact that both serotypes have the same antigenic formula (6,7:m,t-) and other authors showed that they were undisguisable genetically (Robertson et al., 2018). The genome size of these NTS strains varied from 4.6 Mb (Javiana, CFSAN103840) to 5.1 Mb (Oranienburg, CFSAN103872), highlighting the plasticity of NTS genomes (Table 1). A cgMLST analysis showed that these strains were highly divergent but shared at least 3,432 genes out of a total of 4,160 genes of the reference *S*. Enteritidis P125109 (NC_011294) strain used for this cgMLST analysis (Figure 1A; Supplementary Table 1). A minimum spanning tree of the cgMLST analysis showed that they differed in most loci, >78% (Figure 1B). The two Infantis (ST32) differed in at least 136 loci, which was expected since Infantis strain CFSAN103816 lacked the MDR plasmid, indicating that these two strains belonged to very different clones circulating in the same country at the same time.

The closed genomes were further analyzed for the presence of AMR genes using the ResFinder database (Table 1) (Bortolaia et al., 2020). All strains were found to contain *mdsA, mdsB*, and *mdsC* components of the MdsABC complex in the chromosome. MdsABC confers resistance to multiple toxic compounds, including novobiocin, acriflavine, crystal violet, ethidium bromide, methylene blue, rhodamine 6G, tetraphenylphosphonium bromide, benzalkonium bromide, and SDS and oxidative stress-inducing compounds (diamide, H_2_O_2_, Paraquat), and is necessary for colonization of host cells (Nishino et al., 2006; Song et al., 2015). Seven of the strains demonstrated multi-drug resistance with the presence of at least three AMR genes. Aminoglycoside, tetracycline, and sulfonamide resistance genes were detected in seven strains: CFSAN103796 [*aac (6*′*), tetA, aph(3*″*), sul1, aadA1, aac(3)-lva, aph(4)-la*], CFSAN103854 [*aac(6*′*)-Iaa*], CFSAN103872 [*aac(6*′*)-Iaa, aph(3*″*)-lb, aph(6)-ld*], CFSAN103852 [*aac(6*′*)-Iaa, aph(3*″*)-lb, aph(6)-ld*], CFSAN103816 [*aac(6*′*)-Iaa*], CFSAN103862 [*aac(6*′*)-Iaa*, aph(3″)-lb, *aph(6)-ld*], and CFSAN103828 [*aac(6*′*)-Iaa, aph(3*″*)-lb, aph(6)-ld, tetB*] (Table 1).

In clinical *Salmonella* isolates, combined resistance to ampicillin, chloramphenicol, streptomycin, sulfonamides, and tetracycline (ACSSuT) decreased from 8.2% in 1996 to 3.6% in 2009 (Medalla et al., 2013), but other medically relevant antibiotics have become a recent focus of study. Synthetically derived fluoroquinolones were introduced to combat ACSSuT resistance. Three isolates contained the quinolone resistance gene *qnr*_B19_ in plasmids (CFSAN103840, CFSAN103854, and CFSAN103872) of different sizes. Plasmid-mediated quinolone resistance was not identified in retail meat products until 2017 (Sjölund-Karlsson et al., 2010; Tyson et al., 2017). The presence of these small plasmids carrying the *qnr*_B19_ gene is worrisome since they have been identified in other countries in Latin America, such as Peru, Bolivia, Colombia, and Argentina, and appear to be highly mobile among different microorganisms (Tran et al., 2012).

**Figure 2:**
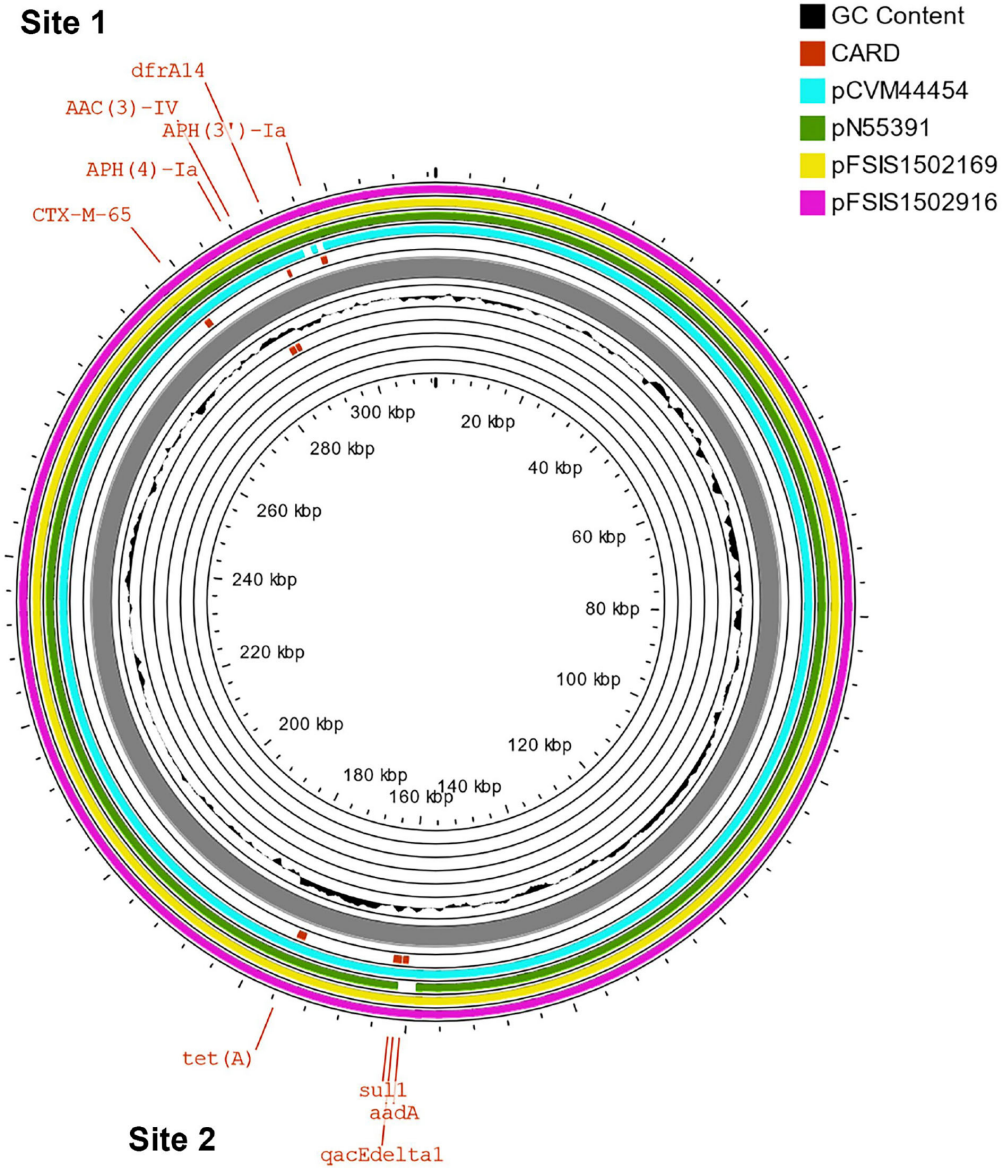
Comparison of *bla*_CTX−M−65_*-*positive plasmids from our study and four other US strains from Tate et al. (2017). Genetic map of the CFSAN103782 plasmid generated with CGView (25). The gray ring is the pCFSAN103796 plasmid. Blue block arrows in the outer circle denote coding regions in the plasmid indicating ORF transcription direction. The G+C content is shown in the middle circle and the deviation from the average G+C content in the innermost circle. BLAST comparisons with four other *blaCTX-M-65*-positive plasmids are shown in cyan (CP016413—strain 2014AM-3028), green (CP016411—strain N55391), yellow (CP016407—strain FSIS1502169), and pink (CP016409—strain FSIS1502916). The two sites containing the AMR genes mentioned in Table 2 are indicated.

The β-lactams, including ampicillin and cephalosporin, are another treatment for invasive salmonellosis, particularly in children. Ceftriaxone resistance increased in the US from 10% in 2002 to 38% in 2010 in chickens, prompting the prohibition of cephalosporin by the FDA in 2012. The extended-spectrum β-lactamase genes *bla*_CTX−M−65_ (CFSAN103796) and *bla*_TEM−1_ (CFSAN103852) were detected in this study on plasmid pCFSA103796 (312,952 bp) and on the chromosome, respectively. An alignment of pCFSA103796 against several *bla*_CTX−M−65−_containing plasmids reported by Tate et al. (2017), showed pCFSAN103796 was highly similar but differed, besides size and SNPs, in two of the main AMR sites from the other reported plasmids (Table 2; Figure 2). There were at least two genes missing in Site 1 that were present in the other plasmids (*floR*, and *fosA*3) while pCFSAN103796 has acquired a *qacEdelta1* gene on Site 2 (Table 2).

**Table 2:** Comparison of *bla*_CTX−M−65_-positive plasmids from our study and four other US strains.

**Strain ID**	**Plasmid size (bp)**	**Plasmid type**	**Genes**		**GenBank accession No**.
			**Site 1**	**Site 2**	
CFSAN103796	312,952	IncFIB	*aph(4)-Ia, aph(3”), aac(3)-Iva, bla_*CTX*−*M*−65_, dfrA14*	*tetA, sul1, aadA1, **qacEdelta1***	CP066336
2014AM-3028	316,160	IncFIB	*aph(4)-Ia, aac(3)-IVa*, *bla*_CTX−M−65_, *floR, dfrA14*	*sul1, tetA, aadA1*	CP016413
N55391	316,814	IncFIB	*aph(4)-Ia*, *aph*(3′*)-Ic, aac(3)-Iva*, *bla*_CTX−M−65_, *floR, dfrA14*	*sul1, tetA*	CP016411
FSIS1502169	323,122	IncFIB	*aph(4)-Ia*, *aph*(3′*)-Ic, aac(3)-IVa*, *bla*_CTX−M−65_, *fosA3, floR, dfrA14*	*sul1, tetA, aadA1*	CP016407
FSIS1502916	322,518	IncFIB	*aph(4)-Ia*, *aph*(3′*)-Ic, aac(3)-IVa*, *bla*_CTX−M−65_, *fosA3, floR, dfrA14*	*sul1, tetA, aadA1*	CP016409

**Figure 3:**
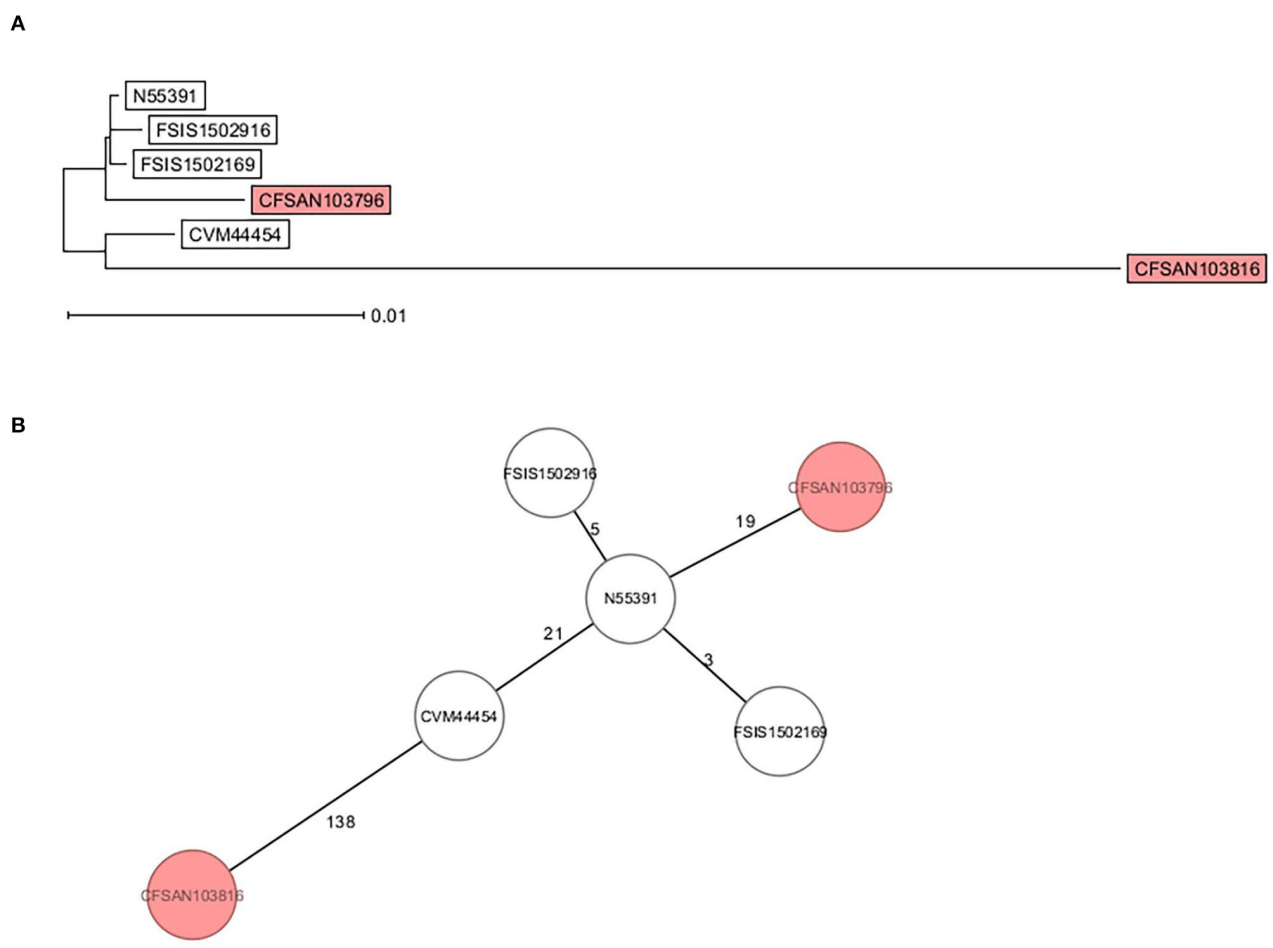
cgMLST analysis of six *S*. Infantis strains showing the divergent evolution of CFSAN103796 from the US strains (2014AM-3028, N55391, FSIS1502169, and FSIS1502916) and the other *S*. Infantis closed genome from Trinidad and Tobago (CFSAN103816). CFSAN103816 genome was used as an outgroup based on 3768 shared genes. **(A)** NJ tree showing the genetic differences among these genomes based on the shared loci rooted in CFSAN103816. **(B)** Minimum spanning tree allowing to visualize the number of loci differences between these genomes. Numbers by lines represent the number of loci differing between strains. The two *S*. Infantis from Trinidad and Tobago are shown in the colored boxes. The lines are not drawn to scale.

Core genome multi-locus sequence type analysis of these 5 Infantis strains (Table 2) and CFSAN103816 (as an outgroup) showed that the 4 strains differed from CFSAN103796 by a minimum of 19 loci differences, further confirming the divergent evolution of this strain from the other ones isolated in the US (Figure 3). Furthermore, Brown et al. (2018) described the presence of *bla*_CTX−M−65−_containing *Salmonella enterica* serotype Infantis isolates in the US that were grouped in the same phylogenic clade as MDR Peruvian isolates (Brown et al., 2018), illustrating the intercontinental spread of this AMR gene. This latter fact highlights the importance of closing the genomes of strains carrying this important AMR gene to track the spreading and evolution of this plasmid through gene acquisitions and SNP accumulations during all these years and in different regions of the world. In turn, this type of analysis could allow establishing the route of dissemination around the world for this strain/plasmid combination as has been done for *S*. Enteritidis (Li et al., 2021). We have additionally detected the presence of the *mcr-9* gene in another strain isolated from this overall study (Maguire et al., 2021). The *mcr-9* gene variant was described in 2019 (Carroll et al., 2019) and appears not to confer colistin resistance in *Salmonella* and *Escherichia coli* (Tyson et al., 2020).

Further analysis of the presence or absence of reported virulence genes for *S. enterica* was determined by an *in silico* approach using the data available in the virulence factor database (VFDB) for that bacterium (Liu et al., 2019) (Table 3). As reported in that table (the complete virulence gene repertoire is available in Supplementary Table 2), all closed *Salmonella* genomes from this study carried both *Salmonella* pathogenicity islands SPI-1 and SPI-2, as reported previously by others (Hoffmann et al., 2016; Lou et al., 2019). SPI-1 contains genes encoding various components of a type III secretion system (T3SS), its regulators, and its secreted effectors (Marcus et al., 2000). SPI-1 enables *S*. *typhimurium* to efficiently penetrate the intestinal epithelium and its role may be limited to the colonization of the gut (Marcus et al., 2000). While SPI-2 contains a second T3SS that is essential for survival within the macrophage and for the establishment of systemic infection (Kombade, 2021). The type III secretion system of SPI-2 is structurally and functionally distinct from that of SPI-1. These two SPIs are necessary for *Salmonella* intestinal epithelial invasion and virulence. An interesting finding was that strain CFSAN103862 serotype Schwarzengrund lacked *prgH, -I, -J, -K* and *orgA* genes in the SPI-1. These genes are required for the secretion of invasion effector proteins and mutant strains lacking these genes were attenuated for invasion in a standard invasion assay of HEp-2 cell monolayers (Fahlen et al., 2000). Some did not carry the *lpf* operon (CFSAN103840—Javiana, and CFSAN103862—Schwarzengrund) which has been shown to be crucial for the attachment of *Salmonella enterica* serovar Typhimurium to murine Peyer's patches (Bäumler et al., 1996). The lack of these many genes in this particular Schwarzengrund strain suggests that it might be less pathogenic to humans since invasion and attachment might be impaired. However proper experiments must be conducted in the future to prove this theory.

**Table 3:** Phenotypic and plasmid classification per strain from this study.

**CFSAN No**.	**Phenotypic AST test results (sensitivity)^a^**	**Plasmid type (plasmid accession No.)^b^**	**Virulence factors^b^**
CFSAN103796	P (S), TE (R), CE (R), AM (R), PH (S), S (R), F (S)	IncFIB (CP066336)	*csg, bcf, lpf*, TTSS (SPI-1 encode), TTSS (SPI-2 encode), TTSS-1 translocated effectors
CFSAN103854	P (S), TE (R), CE (S), AM (R), PH (S), S (S), F (S)	Col-pHAD28 (CP066334)	*csg, bcf, lpf*, TTSS (SPI-1 encode), TTSS (SPI-2 encode), TTSS-1 translocated effectors
CFSAN103872	P (S), TE (R), CE (S), AM (R), PH (S), S (S), F (S)	IncN2 (CP066261)	*csg, bcf, lpf*, TTSS (SPI-1 encode), TTSS (SPI-2 encode), TTSS-1 translocated effectors
CFSAN103852	P (R), TE (R), CE (S), AM (R), PH (S), S (S), F (S)	IncI1-I (CP066329), IncFIB/IncFII (CP066330), IncX1 (CP066331), ColpVC (CP066332)	*gogB, grvA, csg, bcf, lpf*, TTSS (SPI-1 encode), TTSS (SPI-2 encode), TTSS-1 translocated effectors
CFSAN103840	P (S), TE (R), CE (S), AM (R), PH (S), S (S), F (S)	Col-pHAD28 (CP066326)	*csg, bcf*, TTSS (SPI-1 encode), TTSS (SPI-2 encode), TTSS-1 translocated effectors
CFSAN103816	P (S), TE (S), CE (S), AM (R), PH (S), S (S), F (S)	NA	*csg, bcf, lpf*, TTSS (SPI-1 encode), TTSS (SPI-2 encode), TTSS-1 translocated effectors
CFSAN103862	P (S), TE (R), CE (S), AM (R), PH (S), S (S), F (S)	IncFIB/IncFIC (CP066322)	*csg, bcf*, TTSS (SPI-1 encode), TTSS (SPI-2 encode), TTSS-1 translocated effectors
CFSAN103828	P (S), TE (R), CE (S), AM (S), PH (S), S (S), F (S)	IncFIB/IncFII (CP066319), IncX1 (CP066320)	*csg, bcf, lpf*, TTSS (SPI-1 encode), TTSS (SPI-2 encode), TTSS-1 translocated effectors

a *P, penam (amoxicillin–clavulanic acid, 30 μg); TE, tetracycline (doxycycline, 30 μg); CE, cephalo-sporin (ceftriaxone, 30 μg); AM, aminoglycoside (gentamicin, 10 μg, and kanamycin, 30 μg); PH, phenicol (chloramphenicol, 30 μg); S, sulphonamide (sulfamethoxazole–trimethoprim, 23.75 and 1.25 μg); F, fluoroquinolone (ciprofloxacin, 5 μg).*

b *Incompatibility group replicon.*

c *For the complete list, please go to Supplementary Table 2*.

Strain CFSAN103852, a monophasic variant of *S*. Typhimurium was the only one carrying both *gogB* and *grvA* genes. gogB, an anti-inflammatory effector, helps *Salmonella* regulate inflammation-enhanced colonization by limiting tissue damage during infection (Pilar et al., 2012). While grvA, an anti-virulence gene located on the Gifsy-2 phage of *S*. Typhimurium, participates actively in modulating its pathogenicity (Ho and Slauch, 2001). The presence of these genes only in this monophasic *S*. Typhimurium confirms that the pathogenicity of different *Salmonella* serotypes is a complex system and might include redundant systems. Most of these eight strains appear to be pathogenic to humans, although with some differences in virulence profiles.

We further reported the plasmid type and the phenotypic AST test results for the eight strains sequenced in this study. The plasmids carried by these strains contained at least six different incompatibility group replicons, with four of the strains carrying plasmids with an IncFIB incompatibility group replicon (Table 3), which has been reported in many MDR plasmids (Tate et al., 2017).

## Conclusion

The presence of MDR NTS in poultry environments remains a significant global challenge for public health. With the increasing reports of newer variants of AMR genes that confer resistance to diverse types of antibiotics, the need to enact active AMR gene surveillance studies is paramount. With these types of studies, we can not only detect the presence and spread of important AMR genes in poultry, but also identify the strains carrying these AMR genes and their location (whether in the chromosome or plasmids). Here we closed eight genomes of several MDR NTS, determine their ST, serotype, and associated AMR gene profiles, which will be useful for future source tracking, and outbreak investigations in Trinidad and Tobago and worldwide.

## Data Availability Statement

The datasets presented in this study can be found in online repositories. The names of the repository/repositories and accession number(s) can be found in the article/Supplementary Material.

## Author Contributions

MM and NG-E analyzed the data and wrote the manuscript. MM performed laboratory experiments. MM, AK, AA, KG, and NG-E provided materials and critically reviewed the manuscript. All authors contributed to the article and approved the submitted version.

## Funding

This study was supported by funding from the Chief Scientist Challenge Grants Program Proposal Number 2021-200F07A and the FDA Foods Program Intramural Funds. The University of the West Indies, St. Augustine Campus Research and Publication Fund Committee (Research Grant Number 2660-457522) supported the collection of Salmonella from poultry sources in Trinidad and Tobago.

## Conflict of Interest

The authors declare that the research was conducted in the absence of any commercial or financial relationships that could be construed as a potential conflict of interest.

## Publisher's Note

All claims expressed in this article are solely those of the authors and do not necessarily represent those of their affiliated organizations, or those of the publisher, the editors and the reviewers. Any product that may be evaluated in this article, or claim that may be made by its manufacturer, is not guaranteed or endorsed by the publisher.

## References

[B1] Andrews W (1992). Manual of food quality control. 4. Rev. 1. Microbiological analysis. Food And Drug Administration. FAO Food Nutr. Pap. 14, 1–338. 1426189

[B2] Antunes P. Mourao J. Campos J. Peixe L. (2016). Salmonellosis: the role of poultry meat. Clin. Microbiol. Infect. 22, 110–121. 10.1016/j.cmi.2015.12.004 26708671

[B3] Bäumler A.J. Tsolis R. M. Heffron F. (1996). The *Lpf* fimbrial operon mediates adhesion of *Salmonella typhimurium* to murine peyer's patches. Proc. Natl. Acad. Sci. USA 93, 279–283. 10.1073/pnas.93.1.279 8552622 PMC40222

[B4] Bolger A. M. Lohse M. Usadel B. (2014). Trimmomatic: a flexible trimmer for illumina sequence data. Bioinformatics 30, 2114–2120. 10.1093/bioinformatics/btu170 24695404 PMC4103590

[B5] Bortolaia V. Kaas R. S. Ruppe E. Roberts M. C. Schwarz S. Cattoir V.. (2020). Resfinder 4.0 for predictions of phenotypes from genotypes. J. Antimicrob. Chemother. 75, 3491–3500. 10.1093/jac/dkaa345 32780112 PMC7662176

[B6] Brown A. C. Chen J. C. Watkins L. K. F. Campbell D. Folster J. P. Tate H.. (2018). Ctx-M-65 extended-spectrum β-lactamase-producing *Salmonella enterica* serotype infantis, United States. Emerg. Infect. Dis. 24, 2284–2291. 10.3201/eid2412.180500 30457533 PMC6256390

[B7] Carroll L. M. Gaballa A. Guldimann C. Sullivan G. Henderson L. O. Wiedmann M. (2019). Identification of novel mobilized colistin resistance gene *Mcr-9* in a multidrug-resistant, colistin-susceptible *Salmonella enterica* serotype typhimurium isolate. Mbio 10, e00853-19. 10.1128/mBio.00853-19 31064835 PMC6509194

[B8] Darling A. C. E. Mau B. Blattner F. R. Perna N. T. (2004). Mauve: multiple alignment of conserved genomic sequence with rearrangements. Genom. Res. 14, 1394–1403. 10.1101/gr.2289704 15231754 PMC442156

[B9] Deng X. Desai P. T. Den Bakker H. C. Mikoleit M. Tolar B. Trees E.. (2014). Genomic epidemiology of *salmonella enterica* serotype enteritidis based on population structure of prevalent lineages. Emerg. Infect. Dis. 20, 1481–1489. 10.3201/eid2009.131095 25147968 PMC4178404

[B10] Fahlen T. F. Mathur N. Jones B. D. (2000). Identification and characterization of mutants with increased expression of *Hila*, the invasion gene transcriptional activator of Salmonella typhimurium. Fems. Immunol. Med. Microbiol. 28, 25–35. 10.1111/j.1574-695X.2000.tb01453.x 10767604

[B11] Foley S. L. Nayak R. Hanning I. B. Johnson T. J. Han J. Ricke S. C. (2011). Population dynamics of *Salmonella enterica* serotypes in commercial egg and poultry production. Appl. Environ. Microbiol. 77, 4273–4279. 10.1128/AEM.00598-11 21571882 PMC3127710

[B12] Gonzalez-Escalona N. Haendiges J. Miller J. D. Sharma S. K. (2018). Closed genome sequences of two *Clostridium botulinum* strains obtained by nanopore sequencing. Microbiol. Resour. Announc. 7, e01075–18. 10.1128/MRA.01075-18 30533938 PMC6256530

[B13] Gonzalez-Escalona N. Hammack T. S. Russell M. Jacobson A. P. De Jesus A. J. Brown E. W.. (2009). Detection of live *Salmonella* sp. cells in produce by a taqman-based quantitative reverse transcriptase real-time Pcr targeting inva Mrna. Appl. Environ. Microbiol. 75, 3714–3720. 10.1128/AEM.02686-08 19376910 PMC2687310

[B14] Ho T. D. Slauch J. M. (2001). Characterization Of *Grva*, an antivirulence gene on the Gifsy-2 phage in *Salmonella enterica* serovar typhimurium. J. Bacteriol. 183, 611–620. 10.1128/JB.183.2.611-620.2001 11133955 PMC94917

[B15] Hoffmann M. Luo Y. Monday S. R. Gonzalez-Escalona N. Ottesen A. R. Muruvanda T.. (2016). Tracing origins of the *Salmonella* bareilly strain causing a food-borne outbreak in the United States. J. Infect. Dis. 213, 502–508. 10.1093/infdis/jiv297 25995194

[B16] Jackson B. R. Griffin P. M. Cole D. Walsh K. A. Chai S. J. (2013). Outbreak-associated *Salmonella enterica* serotypes and food commodities, United States, 1998–2008. Emerg. Infect. Dis. 19, 1239–1244. 10.3201/eid1908.121511 23876503 PMC3739514

[B17] Khan A. S. Georges K. Rahaman S. Abebe W. Adesiyun A. A. (2021). Characterization Of *Salmonella* isolates recovered from stages of the processing lines at four broiler processing plants in trinidad and tobago. Microorganisms 9, 1048. 10.3390/microorganisms9051048 34068037 PMC8152471

[B18] Khan A. S. Georges K. Rahaman S. Abebe W. Adesiyun A. A. (2022a). Occurrence, risk factors, serotypes, and antimicrobial resistance of *Salmonella* strains isolated from imported fertile hatching eggs, hatcheries, and broiler farms in trinidad and tobago. J. Food Prot. 85, 266–277. 10.4315/JFP-21-236 34706051

[B19] Khan A. S. Pierneef R. E. Gonzalez-Escalona N. Maguire M. Li C. Tyson G. H.. (2022b). Molecular characterization of *Salmonella* detected along the broiler production chain in Trinidad and Tobago. Microorganisms 10, 570. 10.3390/microorganisms10030570 35336145 PMC8955423

[B20] Kolmogorov M. Yuan J. Lin Y. Pevzner P. A. (2019). Assembly of long, error-prone reads using repeat graphs. Nat. Biotechnol. 37, 540–546. 10.1038/s41587-019-0072-8 30936562

[B21] Kombade S. K. N (2021). Pathogenicity Island in Salmonella. London: Intechopen.

[B22] Kuehn B. M (2010). *Salmonella* cases traced to egg producers: findings trigger recall of more than 500 million eggs. JAMA 304, 1316. 10.1001/jama.2010.1330 20858872

[B23] Li S. He Y. Mann D. A. Deng X. (2021). Global spread of *Salmonella* enteritidis *via* centralized sourcing and international trade of poultry breeding stocks. Nat. Commun. 12, 5109. 10.1038/s41467-021-25319-7 34433807 PMC8387372

[B24] Liu B. Zheng D. Jin Q. Chen L. Yang J. (2019). Vfdb 2019: a comparative pathogenomic platform with an interactive web interface. Nucleic. Acids Res. 47, D687–D692. 10.1093/nar/gky1080 30395255 PMC6324032

[B25] Lou L. Zhang P. Piao R. Wang Y. (2019). *Salmonella* pathogenicity island 1 (Spi-1) and its complex regulatory network. Front Cell Infect. Microbiol. 9, 270. 10.3389/fcimb.2019.00270 31428589 PMC6689963

[B26] Maguire M. Khan A. S. Adesiyun A. A. Georges K. Gonzalez-Escalona N. (2021). Closed genome sequence of a *Salmonella Enterica* serotype senftenberg strain carrying the *Mcr-9* gene isolated from broken chicken eggshells in Trinidad and Tobago. Microbiol. Resour. Announc. 10, E0146520. 10.1128/MRA.01465-20 34042489 PMC8201634

[B27] Majowicz S. E. Musto J. Scallan E. Angulo F. J. Kirk M. O'brien S. J.. (2010). The global burden of nontyphoidal *Salmonella* gastroenteritis. Clin. Infect. Dis. 50, 882–889. 10.1086/650733 20158401

[B28] Marcus S. L. Brumell J. H. Pfeifer C. G. Finlay B. B. (2000). *Salmonella* pathogenicity islands: big virulence in small packages. Microbes Infect. 2, 145–156. 10.1016/S1286-4579(00)00273-2 10742687

[B29] Medalla F. Hoekstra R. M. Whichard J. M. Barzilay E. J. Chiller T. M. Joyce K.. (2013). Increase in resistance to ceftriaxone and nonsusceptibility to ciprofloxacin and decrease in multidrug resistance among *Salmonella* strains, United States, 1996-2009. Foodborne Pathog. Dis 10, 302–309. 10.1089/fpd.2012.1336 23464603 PMC6540746

[B30] Nei M. Tajima F. Tateno Y. (1983). Accuracy of estimated phylogenetic trees from molecular data. Ii. Gene Frequency Data. J. Mol. Evol 19, 153–170. 10.1007/BF02300753 6571220

[B31] Nishino K. Latifi T. Groisman E. A. (2006). Virulence and drug resistance roles of multidrug efflux systems of *Salmonella enterica* serovar typhimurium. Mol. Microbiol. 59, 126–141. 10.1111/j.1365-2958.2005.04940.x 16359323

[B32] Pilar A. V. C. Reid-Yu S. A. Cooper C. A. Mulder D. T. Coombes B. K. (2012). Gogb is an anti-inflammatory effector that limits tissue damage during *salmonella* infection through interaction with human Fbxo22 And Skp1. PloS Pathogens 8, E1002773. 10.1371/journal.ppat.1002773 22761574 PMC3386239

[B33] Robertson J. Yoshida C. Kruczkiewicz P. Nadon C. Nichani A. Taboada E. N.. (2018). Comprehensive assessment of the quality of *Salmonella* whole genome sequence data available in public sequence databases using the *Salmonella* in silico typing resource (Sistr). *Microb. Genom*. 4, e000151. 10.1099/mgen.0.000151 PMC5857378 29338812

[B34] Schoeni J. L. Glass K. A. Mcdermott J. L. Wong A. C. (1995). Growth and penetration of *Salmonella enteritidis, Salmonella heidelberg* and *Salmonella typhimurium* in eggs. Int. J. Food Microbiol. 24, 385–396. 10.1016/0168-1605(94)00042-5 7710915

[B35] Sjölund-Karlsson M. Howie R. Rickert R. Krueger A. Tran T. T. Zhao S.. (2010). Plasmid-mediated quinolone resistance among non-typhi *Salmonella enterica* isolates, USA. Emerg. Infect Dis. 16, 1789–1791. 10.3201/eid1611.100464 21029547 PMC3294515

[B36] Song S. Lee B. Yeom J. H. Hwang S. Kang I. Cho J. C.. (2015). Mdsabc-mediated pathway for pathogenicity in *Salmonella enterica* serovar typhimurium. Infect. Immun. 83, 4266–4276. 10.1128/IAI.00653-15 26283336 PMC4598412

[B37] Stothard P. Wishart D. S. (2005). Circular genome visualization and exploration using Cgview. Bioinformatics 21, 537–539. 10.1093/bioinformatics/bti054 15479716

[B38] Tate H. Folster J. P. Hsu C. H. Chen J. Hoffmann M. Li C.. (2017). Comparative analysis of extended-spectrum-beta-lactamase Ctx-M-65-producing *Salmonella enterica* serovar infantis isolates from humans, food animals, and retail chickens in the United States. Antimicrob. Agents Chemother. 61, e00488-17. 10.1128/AAC.00488-17 28483962 PMC5487606

[B39] Tatusova T. Dicuccio M. Badretdin A. Chetvernin V. Nawrocki E. P. Zaslavsky L.. (2016). Ncbi prokaryotic genome annotation pipeline. Nucleic Acids Res. 44, 6614–6624. 10.1093/nar/gkw569 27342282 PMC5001611

[B40] Thomson N. R. Clayton D. J. Windhorst D. Vernikos G. Davidson S. Churcher C.. (2008). Comparative genome analysis of *Salmonella* Enteritidis Pt4 And *Salmonella* Gallinarum 287/91 provides insights into evolutionary and host adaptation pathways. Genome Res. 18, 1624–1637. 10.1101/gr.077404.108 18583645 PMC2556274

[B41] Toro M. Retamal P. Ayers S. Barreto M. Allard M. Brown E. W.. (2016). Whole-genome sequencing analysis of *Salmonella enterica* serovar enteritidis isolates in chile provides insights into possible transmission between gulls, poultry, and humans. Appl. Environ. Microbiol. 82, 6223–6232. 10.1128/AEM.01760-16 27520817 PMC5068155

[B42] Tran T. Andres P. Petroni A. Soler-Bistue A. Albornoz E. Zorreguieta A.. (2012). Small plasmids harboring *qnrb19*: a model for plasmid evolution mediated by site-specific recombination at *Orit* and *Xer* sites. Antimicrob. Agents Chemother. 56, 1821–1827. 10.1128/AAC.06036-11 22290975 PMC3318318

[B43] Tyson G. H. Li C. Hsu C. H. Ayers S. Borenstein S. Mukherjee S.. (2020). The *Mcr-9* gene of *Salmonella* and *Escherichia coli* is not associated with colistin resistance in the United States. Antimicrob. Agents Chemother. 64, e00573-20. 10.1128/AAC.00573-20 32513803 PMC7526823

[B44] Tyson G. H. Tate H. P. Zhao S. Li C. Dessai U. Simmons M.. (2017). Identification of plasmid-mediated quinolone resistance in *Salmonella* isolated from swine ceca and retail pork chops in the United States. Antimicrob. Agents Chemother. 61, e01318-17. 10.1128/AAC.01318-17 28784677 PMC5610501

[B45] Wick R. R. Judd L. M. Gorrie C. L. Holt K. E. (2017). Unicycler: resolving bacterial genome assemblies from short and long sequencing reads. PLoS Comput. Biol. 13, E1005595. 10.1371/journal.pcbi.1005595 28594827 PMC5481147

[B46] Zhang S. Yin Y. Jones M. B. Zhang Z. Deatherage Kaiser B. L. Dinsmore B. A.. (2015). *Salmonella* serotype determination utilizing high-throughput genome sequencing data. J. Clin. Microbiol. 53, 1685–1692. 10.1128/JCM.00323-15 25762776 PMC4400759

